# Action Effects and Task Knowledge: The Influence of Anticipatory Priming on the Identification of Task-Related Stimuli in Experts

**DOI:** 10.1371/journal.pone.0156928

**Published:** 2016-06-07

**Authors:** William M. Land

**Affiliations:** Department of Kinesiology, Health, & Nutrition, University of Texas at San Antonio, San Antonio, Texas, United States of America; Leiden University, NETHERLANDS

## Abstract

The purpose of the present study was to examine the extent to which anticipation of an action’s perceptual effect primes identification of task-related stimuli. Specifically, skilled (*n* = 16) and novice (*n* = 24) tennis players performed a choice-reaction time (CRT) test in which they identified whether the presented stimulus was a picture of a baseball bat or tennis racket. Following their response, auditory feedback associated with either baseball or tennis was presented. The CRT test was performed in blocks in which participants predictably received the baseball sound or tennis sound irrespective of which stimulus picture was displayed. Results indicated that skilled tennis players responded quicker to tennis stimuli when the response was predictably followed by the tennis auditory effect compared to the baseball auditory effect. These findings imply that, within an individual’s area of expertise, domain-relevant knowledge is primed by anticipation of an action’s perceptual effect, thus allowing the cognitive system to more quickly identify environmental information. This finding provides a more complete picture of the influence that anticipation can have on the cognitive-motor system. No differences existed for novices.

## Introduction

There is growing evidence that the perceptual-cognitive skill of anticipation is a fundamental control and performance variable within the domain of human performance. The ability to accurately anticipate and predict forthcoming actions and their effects has been shown to underlie both cognitive and motor performance across a range of domains and skill sets [1, 2]. To this extent, anticipatory skills have been identified as an important distinguishing factor between skilled and less skilled performers, even when compared to anthropometric and physiological factors [3, 4]. Given the importance and broad ranging influence of anticipation, further examination of the cognitive mechanisms supporting the benefits associated with anticipation is important for both motor control and sport science research.

From a motor control perspective, anticipation has been suggested to play an essential role during the initial stages of action control [1, 5, 6]. Specifically, the planning and control of goal-directed action is considered to be based on the anticipation of an action’s intended outcome [1]. Perhaps the best example of this effect comes from the work on anticipatory motor control (e.g., see [7–9]). For instance, Rosenbaum et al. [9] found that participants grasped objects differently depending on the anticipated future placement of the object (e.g., end-state comfort effect). Such findings have been taken as evidence that the anticipation of future movement goals influence the control and coordination of actions (e.g., [10–15]).

Similarly, anticipatory skills have also been identified to underlie a number of important aspects of sport performance [16]. For instance, the research on focus of attention highlights the robust influence of anticipation on skilled performance (e.g., [17–21]). Specifically, an external focus on the perceptual effects of a movement (e.g., anticipated future flight of a tennis serve) has been shown to result in superior performance compared to an internal focus of attention directed towards the movement itself (see [21]). The performance advantage associated with adopting an external focus has been shown to generalize across a variety of tasks such as baseball batting [18, 22], basketball free throws [23], golf putting [20, 24], tennis serves [25], and performance on ski simulators [26].

The underlying mechanism accounting for the performance benefits linked to anticipating forthcoming external effects has been associated with principles derived from ideomotor theories of voluntary action [20]. Primarily, ideomotor accounts (e.g., see [27–30]) suggest that actions are guided by the representation of the action’s sensory feedback, especially feedback external to the performer [5, 31]. Developed through learning contingencies between movements and their effects, learners are believed to acquire integrated sensorimotor representations, which contain knowledge regarding both the efferences (i.e., cognitive output signals to the motor system) and their reafferances (i.e., sensory feedback produced by the action) for a particular action [1]. Importantly, the encoding of motor commands and their reafferances within these sensorimotor representations is stated to be bi-directional in so far that the anticipation of an action’s reafferences can cue forth the motor commands that bring about the desired motor execution [32]. Consequently, the anticipation of an action’s external sensory effects is suggested to prime and cue the action itself. With respect to an external focus, the associated benefits are based on the anticipation of an action’s effect brought about by focusing attention externally [20].

Evidence to support this theoretical account has come primarily from reaction-time experiments [32–36]. For instance, Elsner and Hommel [32] had participants produce key presses that resulted in simple auditory effects (tones of varying pitch) during an initial learning phase. In a subsequent transfer phase, the auditory tones were used as imperative stimuli in a choice-reaction time task. If actions and their effects are bidirectionally linked during learning, then during a transfer phase, the presentation of the effect tone should act to prime the associated action (i.e., keypress). The results of Elsner and Hommel’s study supported this assertion, as reaction times were faster when the response was cued by its former effect tone than when cued by an effect tone associated with an alternative response. These findings suggest that learned action effects can act as effective retrieval cues or primes of the associated motor response.

Interestingly, the influence of action effects has been shown to influence performance both endogenously and exogenously. The above Elsner and Hommel [32] study represents exogenous action priming by way of presenting the action effect as a direct stimulus prior to a motor response (i.e., cue-driven response). In contrast, endogenous activation occurs as a result of merely anticipating an intended action effect. For example, Kunde ([37], Experiment 2) had participants respond with a forceful or soft key press in response to either a red or green stimulus presented on a computer screen. Following the response, an auditory tone effect was presented that varied in intensity (i.e., loud or soft). For the participants, a soft key press was associated through learning with producing a soft tone, whereas the firm key press was associated with producing a loud response tone. Participants performed the choice-reaction test in blocks in which irrespective of the key press, the participants would predictably always hear either the loud or soft tone following the key press. Results indicated that key presses were initiated faster when they were predictably followed by their associated response tone (i.e., soft key presses were initiated quicker during blocks in which soft tones predictably followed the response). Similarly, Pfordresher [38, 39] demonstrated that producing musical sequences on a piano can be disrupted when the pitch of the notes are altered from what is anticipated. In both cases, the evidence suggests that the anticipation of a given action effect plays a generative function for the selection and execution of the action associated with the effect [5]. Instances where anticipation of a given effect is not aligned with the action to be performed, performance suffers [40].

The above research provides overwhelming support for the influence of anticipation on motor control and skill execution. However, it may be an over-simplification to assume that the anticipation of an action’s effect only functions to prime and cue a motor response (e.g., [5]). It may be more plausible to assume that anticipation of an action’s effect cues not only the underlying motor code, but also the accompanying domain knowledge associated with the skill. More specifically, the sensorimotor representations, which contain the efferent and afferent codes, exist within a larger hierarchical representation network consisting of related task-specific knowledge [1, 41]. A more holistic activation of the overall task-specific knowledge representations, rather than simply the motor codes, would give the cognitive system quicker access to acquired knowledge [42, 43]. The priming of task-specific knowledge representations may help the system more effectively adapt to changing situations, potentially through quicker identification of task-related environmental information. To this extent, the activation of knowledge through priming can facilitate the perception of conceptually related information [43, 44]

Initial support for this assumption comes from work on perceptual resonance, that is, the modulation of perception by action [45, 46]. Evidence suggests that a planned or concurrent action can influence the perceptual sensitivity of movement relevant stimuli (see [47–50]). Specifically, research on perceptual assimilation indicates planning for an action can enhance the detection and identification of action-related stimuli. For example, Craighero et al. [47] found evidence that preparation to grasp an object facilitated processing of visual stimuli relevant to the object. Furthermore, Fagioli, Hommel, and Schubotz [51] suggest that planning an action biases the perceptual system towards task relevant feature dimensions thus facilitating the detection and discrimination of action-related information. Given that anticipation of an intended outcome underlies the planning and control of action, it appears plausible that it may also facilitate the detection of task-related stimuli.

To further clarify the influence of anticipation, the present study examined whether anticipation of an action’s effect (i.e., endogenous priming) influences the identification and detection of task-related stimuli. Hence, we propose an effect-induced knowledge priming hypothesis, such that anticipation of an action effect primes task-related domain knowledge. This assumption builds on previous work which indicates that anticipation of an action effect cues an associated motor response [1, 5]. However, here we asked whether the anticipation of an action effect facilitates the identification of task-related stimuli, rather than priming of a motor response. Critically, the extent to which anticipation influences action control, and presumably the identification and detection of task-related stimuli, is dependent upon knowledge about the relationship between the action and its effect [1]. Thus, previous experience and skill level underlie the effect of anticipation. As such, a priming effect should only be observed for an individual’s particular area of domain expertise.

In the present study, novice and skilled tennis players performed a choice-reaction time (CRT) test in which they indicated whether the presented stimulus was related to the task of baseball or tennis. Each response led to the presentation of a sound effect associated with either baseball or tennis. Importantly, the CRT test was performed in blocks in which the auditory feedback following the response was predictably associated with either baseball or tennis regardless of the stimulus presented. Given this predictability in the auditory feedback, participants were able to anticipate the forthcoming feedback following a CRT response. Consistent with the effect-induced knowledge priming hypothesis, we expected that skilled tennis players would have substantially shorter response latencies when identifying the tennis picture during blocks in which the response was predictably followed by the congruent tennis auditory feedback compared to the incongruent feedback (i.e., baseball auditory feedback). In contrast, given the participants’ lack of experience with the domain of baseball, no effect of priming was expected for the identification of baseball stimuli regardless of auditory feedback congruency. Furthermore, given that the novice participants were neither familiar with tennis nor baseball, we predicted no effect of congruency on reaction times for trials in which the auditory feedback was congruent or incongruent to the stimuli. Overall, such findings would indicate that anticipation of a perceptual effect can facilitate the identification of task-related information given sufficient domain experience.

## Method

### Participants

Participants consisted of novice (*n* = 24) and skilled tennis players (*n* = 16). Novice participants (9 male, 14 female) ranged in age from 20 to 38 (*M* = 25.7, *SD* = 3.99), and reported little to no previous tennis experience. Skilled participants (11 male, 5 female) ranged in age from 18 to 59 (*M* = 31.5, *SD* = 15.14), and reported an average of 13.8 years of tennis experience (*SD* = 6.48). Additionally, all participants (both novice and skilled tennis players) reported little to no experience or knowledge of baseball. Skilled participants were recruited from a private tennis club in Germany, whereas novice participants were recruited from a University in Germany. The experimental procedures were approved by the ethics committee at Bielefeld University, and conformed to the Declaration of Helsinki. Written informed consent was obtained from all participants prior to participation in the study. Participants received 5€ for participation.

### Apparatus and Stimuli

**Fig 1:**
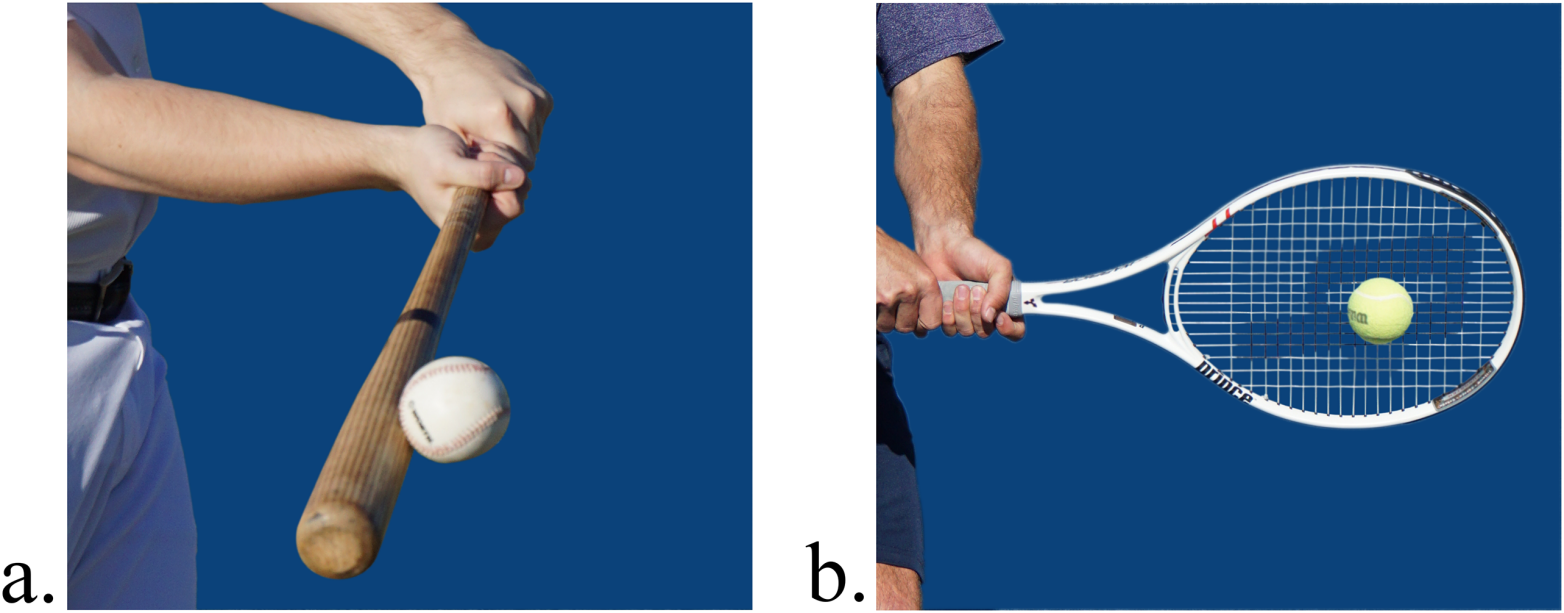
Stimuli. Visual stimuli depicting the (a) baseball task and (b) tennis task.

The picture stimuli for the CRT trials are presented in Fig 1. The picture stimuli depicted the moment of contact between the ball and the sport implement (i.e., baseball bat or tennis racket). The background of each picture was removed and replaced with a dark blue solid color to reduce distraction from irrelevant background information. The stimuli were presented centrally on a monitor, and were displayed at a size of 16.5 × 16.5cm (11.8° × 11.8° of visual angle from a distance of 80cm). A white fixation cross (3 × 3cm) on a black background was presented prior to each stimulus picture.

Following a response, either a baseball or tennis sound was presented to the participant. The baseball audio consisted of the sound of a bat making contact with a baseball. Likewise, the tennis audio consisted of a racket making contact with a tennis ball. The content of the sound files were selected to match the action displayed in the picture stimuli. Each sound file was presented through headphones worn by the participant at a comfortable loudness (60 dB). Both sound files were 30ms in duration.

The CRT task was administered on an IBM compatible computer with a 19-inch VGA-Display using the software Presentation^®^ (version 14.1; http://www.neurobs.com). The software controlled the presentation of the visual and auditory stimuli, as well as measured the reaction time of the responses (accuracy < 1ms). Participants responded to the stimuli through a response device that consisted of two buttons located 18cm apart and was located directly in front of the participants.

### Procedures

Participants were seated in front of a computer screen (at a distance of 80cm), and were instructed to determine whether the presented stimuli related to either baseball or tennis. Furthermore, they were instructed that each response would lead to a certain sound. Participants responded by pressing one of two external buttons on a response device corresponding with a choice of baseball or tennis. Button response assignment was counterbalanced across participants. Participants were instructed to respond to the stimuli as fast and accurately as possible.

**Fig 2:**
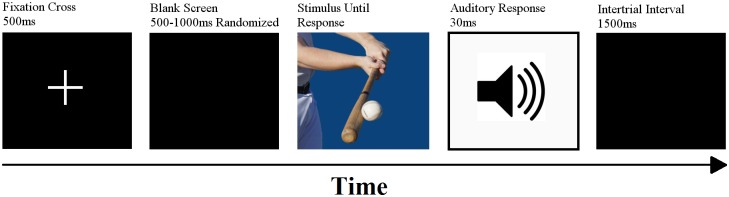
Order of CRT Stimuli. Example of a CRT trial depicting the order and time length of each element of the trial.

Each trial started with a fixation cross (500ms) followed by a blank black screen (500ms-1000ms randomized) and then the picture stimulus (until a response was detected). Immediately following the response, the appropriate sound based on the condition was emitted. After an inter-trail interval of 1500ms, the next trial began (cf. Fig 2). Incorrect responses resulted in a visually presented feedback error (“Fehler”: the German word for mistake).

Participants began the experiment by completing 10 randomized practice trials. For the practice trials, the auditory feedback always corresponded to the picture stimuli (e.g., tennis picture and tennis sound). Following, participants performed the CRT task across 6 blocks consisting of 50 trials each (randomized between baseball and tennis stimuli). For each block, participants always received the same auditory feedback for all 50 trials regardless of the stimulus or response. Each block alternated between the baseball and tennis sound. Consequently, each block was comprised of trials that were either congruent or incongruent in terms of the picture stimuli matching with the auditory response. For instance, in a block in which the baseball sound always followed the response, a trial in which the baseball picture was presented would be considered congruent. Thus, a trial was considered congruent if the picture stimulus (baseball or tennis) corresponded with the auditory feedback from the same sport (i.e., baseball or tennis feedback). Trials in which the stimulus picture was followed by auditory feedback from a different sport were considered incongruent. Participants were informed prior to each block regarding which auditory feedback sound would follow their responses.

### Data Analyses

Reaction times (RTs) were filtered for outliers with values below (200ms) and above (1000ms) excluded from all analyses (1.9%) [52]. Wrong answers were also subsequently discarded (1.44%). Mean RTs were calculated for each participant according to both sport type and auditory feedback congruency. For data analysis, the first ten trials of each block were discarded as warm-up trials. The filtered RTs were subsequently examined via a 2 × 2 × 2 mixed-models analysis of variance (ANOVA) with sport (tennis, baseball) and auditory congruency (congruent, incongruent) as within-subjects factors, and skill level (skilled vs. novice tennis players) as a between-subjects factor. For post-hoc analysis, paired t-tests were conducted employing a Bonferroni correction (α = .0125) to account for the inflation of type I errors. To ensure that the adopted measure of central tendency did not influence our results, we computed the same analyses with medians rather than means. The results, however, revealed essentially the same statistical findings as with the means. Additionally, error rates were also examined via the mixed-model ANOVA.

## Results

### Response Times

**Fig 3:**
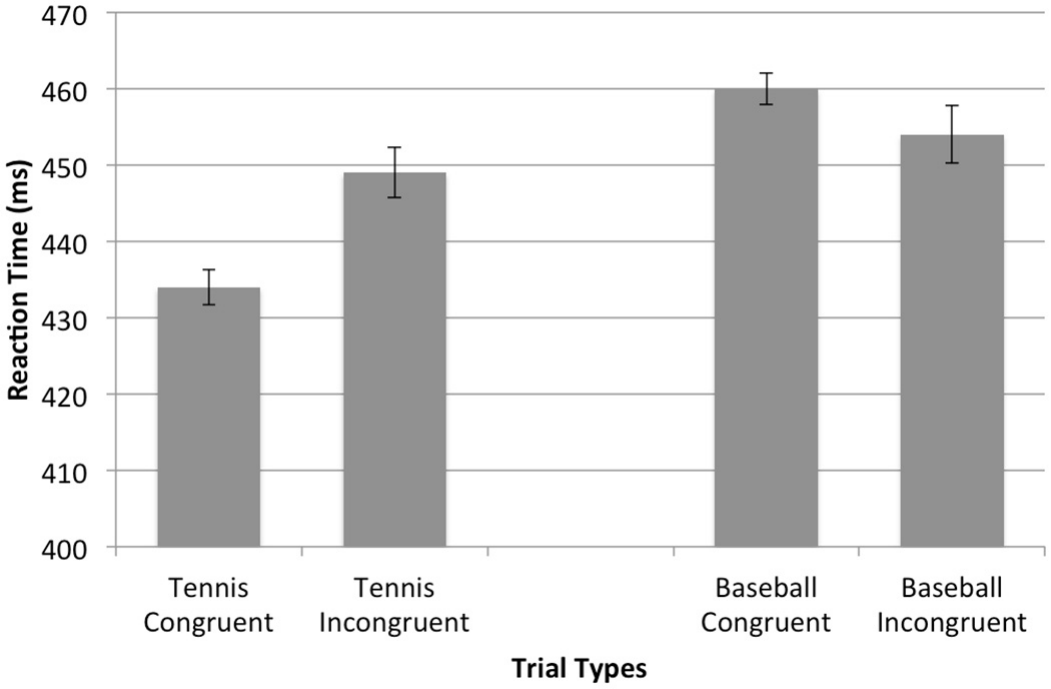
Skilled Reaction Times. Mean reaction times for skilled tennis players across both congruent and incongruent trials for tennis and baseball. Error bars represent standard error.

**Fig 4:**
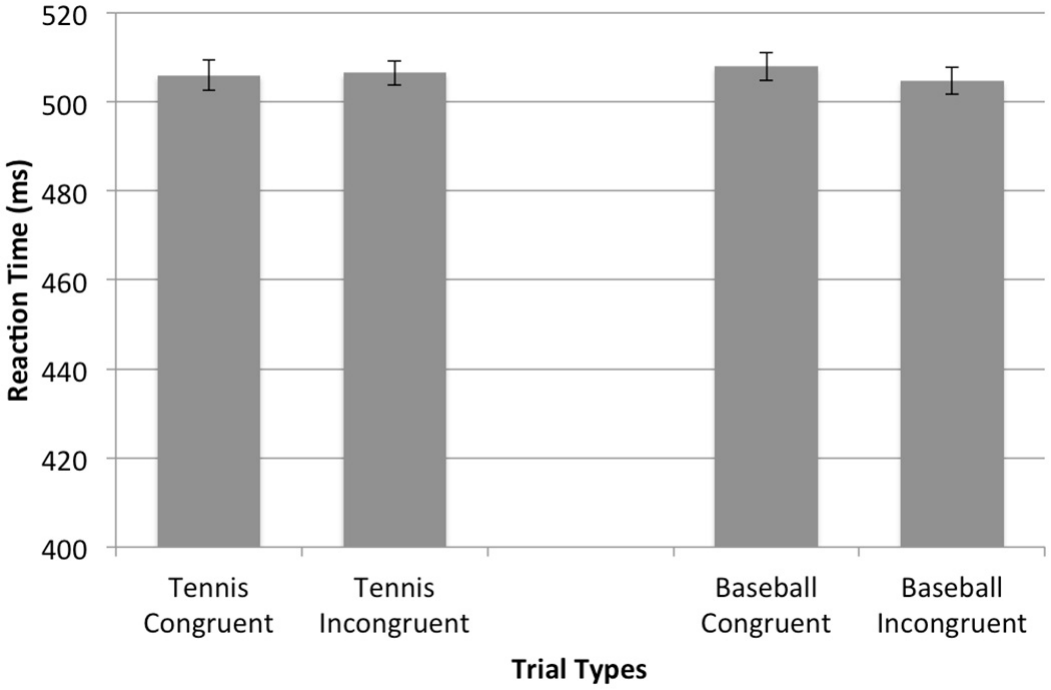
Novice Reaction Times. Mean reaction times for novice tennis players across both congruent and incongruent trials for tennis and baseball. Error bars represent standard error.

Mean response times for skilled and novice tennis players across both corresponding and non-corresponding trials for tennis and baseball are presented in Figs 3 & 4, respectively. Additionally, results of the mixed-model ANOVA are presented in Table 1. The mixed-model ANOVA indicated a significant main effect of sport, *F*(1,38) = 5.236, *p* = .028, *η*^2^ = .07. Responses to the tennis picture were faster than responses to the baseball picture, 473 ms (*SEM* = 16.03) vs 481 ms (*SEM* = 16.69). More importantly, however, the mixed-model ANOVA indicated a significant sport × skill level interaction, *F*(1,38) = 4.957, *p* = .032, *η*^2^ = .06. To further clarify this interaction, bonferroni corrected pair-wise comparisons revealed that skilled tennis players were significantly faster at responding to the tennis stimuli (441 ms, *SEM* = 25.86) compared to the baseball stimuli (457 ms, *SEM* = 24.83), *p* = .006. In contrast, no significant differences existed between the response latencies of novice tennis players in responding to the tennis (506 ms, *SEM* = 21.12) or baseball stimuli (506 ms, *SEM* = 20.27), *p* = .96. This finding is consistent with previous work that suggests that task familiarity can facilitate response selection and reaction times given multiple response alternatives (e.g., [53, 54]).

**Table 1:** Mixed model ANOVA Results for Response Times.

Effect	Degrees of freedom	*F*	*p*	*η*^2^
A. Sport	1, 38	5.236	.028	.066
B. Congruence	1, 38	0.432	.515	.002
C. Skill Level	1, 38	3.072	.088	.075
D. A × B	1, 38	8.449	.006	.038
E. A × C	1, 38	4.957	.032	.062
F. B × C	1, 38	1.797	.188	.007
G. A × B × C	1, 38	4.224	.047	.019

Next, the mixed-model ANOVA indicated a significant sport × auditory congruency interaction, *F*(1,38) = 8.449, *p* = .006, *η*^2^ = .038. However, more importantly, this interaction is further clarified by a signification sport × auditory congruency × skill level interaction, *F*(1,38) = 4.224, *p* = .047, *η*^2^ = .019. To further elucidate this interaction, separate sport × auditory congruency repeated-measures ANOVA were performed for both novice and skilled tennis players. For skilled tennis players, results of the RM ANOVA indicated a significant main effect of sport, *F*(1,15) = 22.92, *p* < .001, *η*^2^ = .273. More importantly, a significant sport × auditory congruency interaction was observed, *F*(1,15) = 9.211, *p* = .008, *η*^2^ = .118. Follow-up pair-wise comparisons revealed that skilled tennis players were significantly faster responding to the tennis stimuli when the response was followed by the tennis auditory effect (congruent mapping, 434 ms, *SEM* = 11.4) compared to the baseball auditory effect (incongruent mapping, 449 ms, *SEM* = 11.5), *t*(15) = -2.992, *p* = .009. Furthermore, participants were also significantly faster responding to tennis congruent CRT trials (434 ms, *SEM* = 11.4) compared to baseball congruent CRT trials (460 ms, *SEM* = 11.9), *t*(15) = 9.794, *p* < .001, while no significant differences were observed between tennis incongruent (449 ms, *SEM* = 11.5) and baseball incongruent CRT trials (454 ms, *SEM* = 11.1), *t*(15) = .880, *p* = .393. Lastly, an effect of congruency was not observed for responses to the baseball stimuli. No significant differences existed between the baseball congruent (460 ms, *SEM* = 11.9) and baseball incongruent (454 ms, *SEM* = 11.1) CRT trials, *t*(15) = 1.223, *p* = .24. For the novice tennis players, a separate RM ANOVA indicated no significant main effects or interactions, all *p’s* > .05.

### Error Rates

Table 2 presents the mean (SD) percentages of errors for congruent and incongruent auditory trials for both tennis and baseball across novice and skilled participants. Results of a mixed model ANOVA with sport and auditory congruency as within-subjects factors and skill level as a between-subjects factor revealed a significant main effect of congruency, *F*(1,38) = 6.00, *p* = .019, *η*^2^ = .061. Although rare, participants made less response errors during trials in which the required response was congruent to the auditory feedback (1.1%, *SEM* = .163) compared to trials in which the required response was incongruent to the anticipated auditory feedback (1.8%, *SEM* = .288). These findings taken together with the corresponding pattern of response times, especially those of the skilled participants, suggests that the benefit associated with effect-induced priming is likely not related to a speed-accuracy tradeoff given that the congruent trials, which were associated with faster RTs, were not related to increased error rates.

**Table 2:** Percentages of Error Trials.

	Congruent Feedback		Incongruent Feedback	
	Tennis	Baseball	Tennis	Baseball
**Skilled**	1.04 (1.19)	1.35 (1.25)	2.08 (2.06)	1.67 (1.92)
**Novice**	1.04 (1.46)	.97 (1.47)	1.46 (1.79)	2.01 (2.55)

## General Discussion

The primary purpose of the present study was to examine the extent to which anticipation of an action’s perceptual effect influences task-related stimulus identification. Specifically, we compared response times toward stimuli predictably followed by either congruent or incongruent action effects. According to our proposed effect-induced knowledge priming hypothesis, we predicted that the anticipation of an action’s perceptual effect would act to endogenously prime task-related domain knowledge. As a consequence, identification of task-related stimuli would be facilitated and result in faster reaction times. Results supported this assumption, as the reaction times of skilled tennis players were significantly faster when responding to tennis stimuli followed by a congruent action effect (i.e., sound of hitting a tennis ball) compared to an incongruent action effect (i.e., sound of hitting a baseball). These findings suggest that the anticipation of the forthcoming action effect activated domain-related knowledge prior to the response, which aided stimulus identification and resulted in reduced response latencies.

As indicated by ideomotor accounts of voluntary action (e.g., [27, 30, 32]), anticipation of an action’s effect is stated to prime sensorimotor representations comprised of action (i.e., efferent) and effect (i.e., reafferent) codes [1]. Furthermore, these sensorimotor representations are integrated into larger representational networks comprising of task knowledge (e.g., context information, verbal descriptions, and declarative task information). In addition to the present results, support for this integration comes from research that indicates that sensorimotor representations entertain associative links to other task related elements such as overt and covert speech [55, 56] and affect representations [57]. For instance, there is evidence that verbal labels can become associated with sensorimotor representations, and functionally act as a retrieval cue for a planned action [1]. Likewise, Beckers et al. [57] observed that actions become associated not only with the effects they produce, but also the affective valence of the consequence. In the case of the present study, the results suggest that sensorimotor representations also become connected to task-relevant knowledge, and that primed activation of these sensorimotor representations via anticipation spreads activation to associated task knowledge. As such, the shared activation of domain knowledge increases activation potential, and results in faster access to necessary stored information allowing for quicker identification of task-related stimuli [43]. Furthermore, analysis of error rates suggests that the anticipation of incongruent action effects can increase the failure to accurately identify stimuli.

Importantly, the benefit of anticipation on effect-induced knowledge priming only extends to stimuli within the participants’ domain of expertise. More precisely, skilled tennis players only responded faster to the tennis picture when it was primed via anticipation of the subsequent tennis auditory feedback. A similar congruence effect was not observed when the tennis players responded to the baseball stimuli when followed by the baseball auditory effect. This is not surprising given the lack of baseball knowledge amongst the participants. In fact, there were no significant differences between tennis incongruent CRT trials and baseball congruent or incongruent CRT trials.

Given this pattern of findings (see Fig 3), it is important to consider whether the congruent tennis CRT trials reflected a benefit to information processing or whether the incongruent tennis CRT trials reflected an interference or disadvantage to RT performance. To delineate this distinction, the RT performance when responding to the baseball CRT trials can be considered as a neutral or control condition due to the lack of baseball experience by the skilled tennis players. Thus, no priming would have taken place during trials in which the tennis players identified the baseball stimuli. Importantly, there was no difference between the baseball congruent, baseball incongruent, and tennis incongruent trials. Had the tennis incongruent trials resulted in slower RTs compared to the baseball congruent or incongruent trials, then a case could be made that incongruency within a participant’s domain of expertise can have a debilitative effect. However, the lack of difference between tennis incongruent and the baseball trials suggests there was no negative effect. Thus, given that the tennis congruent condition was significantly faster than all other conditions, including the baseball control conditions, points to a benefit of congruency rather than an interference of incongruency. However, future research needs to validate this interpretation. Specifically, future research should include a control condition in which no auditory feedback is given (i.e., thus no anticipatory priming). This would allow for a direct comparison of the effect of congruent and incongruent priming on RT performance.

Additionally, it can also be concluded that the reduced response latencies for the tennis stimuli during blocks with tennis auditory feedback is likely not a byproduct of increased motivational or attentional factors. This potential explanation can be discounted because any increased motivation or alertness during this block would have also produced faster responses to the baseball picture. However, this was not the case.

Additional support of the impact that skill level has on the occurrence of effect-induced knowledge priming is observed from the findings, or lack thereof, from the novice participants. As predicted, no differences were observed in reaction times regardless of sport or auditory congruence. As such, the anticipation of a given perceptual action effect had no influence on the speed at which individuals could distinguish between stimuli from different domains. This finding is consistent with and reinforces the findings from the skilled participant data whereby the skilled tennis players revealed no differences in reaction times to baseball stimuli regardless of anticipatory priming. Thus, without knowledge and experience with a particular domain, no influence of priming can take place.

The ideomotor account, in particular Elsner and Hommel’s [32] two-stage model of voluntary action, provides a clear explanation for the observed differences between skill levels with respect to the occurrence of effect-induced priming. Specifically, Stage 1 of the model consists of developing learned contingencies between an action and its effect, and presumably related task knowledge. Given that novices do not have the learned association between a movement and its effect, the anticipation of these effects does not lead to priming of the associated movement (Stage 2 of the model). In other words, the ability to prime action, and thus associated task knowledge, is dependent on acquiring these underlying associations. As such, anticipation of the forthcoming action effect has no influence on the ability of novice participants to discern between task-related and task-unrelated stimuli.

From an ideomotor perspective, the influence of action effects on human behavior has traditionally centered around the impact of such effects on movement production. With respect to information processing stage theory, Kunde et al. [5] found that anticipation of an action effect has an impact on all phases of response production (i.e., response selection, response initiation, and response execution). In the current study, however, we suggest that anticipation of an action effect may also influence earlier stages of information processing, namely stimulus identification. More specifically, our effect-induced knowledge priming hypothesis proposes that primed activation of sensorimotor representations spreads activation to associated task knowledge. This primed activation of task knowledge facilitates the speed and accessibility to stored information, through an increased activation potential (reduced response threshold), which can facilitate the perception and identification of task-related stimuli [43, 44]. Furthermore, it is unlikely that the reduced response latencies observed for the skilled participants on the congruent tennis trials is due to an influence on the response production stages (i.e., response selection, response initiation, or response execution) given that there should be no inherent relationship between tennis or baseball and the pressing of a left or right response button. Similarly, Bläsing et al [58] concluded that action congruent primes influenced RT performance primarily during the cognitive processing stages rather than the motor execution stages. However, future research is needed to fully explore the processing stages accounting for the current findings.

A potential limitation of the present study lies with the methodology used to allow for anticipation of a specific perceptual effect. Specifically, the current study had participants perform the CRT task in blocks in which the auditory feedback was consistent regardless of CRT response. This consistency allowed the auditory feedback to serve as a reliable mental cue for the CRT response. However, a potential confound exists in that the response feedback from one trial may exert an influence on the subsequent trial (cf. [59], for an overview). While this influence cannot be ruled out in the present study, Kunde et al. ([5], Experiment 1), investigated whether the influence of preceding trials in CRT tasks with blocked compatibility effects exert an influence on subsequent trials. Incorporating an intertrial interval of 1000ms, their results indicated that the previous trial had little to no influence on the subsequent trial. Given these findings, and that the present study incorporated a longer intertrial interval of 1500ms, we assume that the obtained results do not reflect the influence of preceding trials. However, future research should attempt to experimentally control for the possibility of intertrial effects.

## Conclusion

The anticipation of an action’s effect has previously been shown to play a generative function for the selection and execution of movement [5]. The present study extends this line of work by highlighting that effect anticipations also create an internal readiness to respond to task-related stimuli. Our findings extend the knowledge regarding the range of dimensions by which anticipated action effects can influence human behavior. To this extent, anticipatory priming prepares both the cognitive and motor system for responding to task demands. Specifically, according to our proposed effect-induced knowledge priming hypothesis, task-related information is primed through effect anticipation thus allowing for faster identification of information related to the task at hand. Furthermore, the extent of effect-induced knowledge priming is mediated by domain expertise, thus highlighting the integration of sensorimotor representations with task relevant knowledge.

From a practical standpoint, it would appear logical that the preparation to act would prime not only the corresponding motor action, but also prepare the cognitive system to attend to relevant task related information. Such preparation would allow the system to more effectively adapt and respond to new and changing environmental information. Thus, the ability to quickly identify between related and unrelated information is critical for flexible and efficient performance. However, future research is needed to clarify the extent of the priming effect. Specifically, to what extent must the stimuli be associated with the anticipated action effect? In the current study, the content of the auditory feedback temporally matched with the visually presented stimuli (e.g., both the visual and auditory stimuli related to the tennis racquet-ball contact). It would be of interest to determine whether the anticipated effect must align temporally to the stimuli for which the system must discern (e.g., whether auditory feedback of the bat-ball contact could help RT performance to stimuli that occurs prior to bat-ball contact). Notwithstanding these needed clarifications, the present study clearly shows that the anticipation of an action’s perceptual effect influences task-related stimulus identification. Our findings, together with prior research on action-effect priming of motor actions [32, 37], provides a more holistic account of the influence that anticipation can have on both the cognitive and motor system in preparation for action.
